# Membrane potential stimulates ADP import and ATP export by the mitochondrial ADP/ATP carrier due to its positively charged binding site

**DOI:** 10.1126/sciadv.adp7725

**Published:** 2024-11-01

**Authors:** Vasiliki Mavridou, Martin S. King, Andre Bazzone, Roger Springett, Edmund R. S. Kunji

**Affiliations:** ^1^ MRC Mitochondrial Biology Unit, University of Cambridge, Cambridge Biomedical Campus, Keith Peters Building, Cambridge CB2 0XY, UK.; ^2^ Nanion Technologies GmbH, Ganghoferstrasse 70A, D-80339 Munich, Germany.; ^3^ CellSpex, Kettering, Northamptonshire NN14 6GX, UK.

## Abstract

The mitochondrial adenosine 5′-diphosphate (ADP)/adenosine 5′-triphosphate (ATP) carrier imports ADP into the mitochondrion and exports ATP to the cell. Here, we demonstrate that 3.3 positive charges are translocated with the negatively charged substrate in each transport step. They can be assigned to three positively charged residues of the central substrate-binding site and two asparagine/arginine pairs. In this way, the membrane potential stimulates not only the ATP^4−^ export step, as a net −0.7 charge is transported, but also the ADP^3−^ import step, as a net +0.3 charge is transported with the electric field. These positive charge movements also inhibit the import of ATP and export of ADP in the presence of a membrane potential, allowing these nucleotides to be maintained at high concentrations in the cytosol and mitochondrial matrix to drive the hydrolysis and synthesis of ATP, respectively. Thus, this is the mechanism by which the membrane potential drives adenine nucleotide exchange with high directional fluxes to fuel the cellular processes.

## Teaser

Reorientation of a positively charged binding site drives adenine nucleotide exchange in the presence of a membrane potential.

## INTRODUCTION

The mitochondrial adenosine 5′-diphosphate (ADP)/adenosine 5′-triphosphate (ATP) carrier, also called adenine nucleotide translocase, carries out one of the most prolific transport steps, sustaining all the energy-requiring processes of the cell (*1*, *2*). As an essential part of the eucaryotic oxidative phosphorylation, the carrier imports cytosolic ADP into the mitochondrial matrix for its conversion to ATP by the ATP synthase and exports the synthesized ATP to the intermembrane space, which is confluent with the cytosol, to replenish cytosolic ATP pools (*1*–*3*). Collectively, these carriers transport approximately our own body weight in ADP and ATP every day across the mitochondrial inner membrane, recycling each ATP molecule every minute (*4*, *5*). The mitochondrial ADP/ATP carrier is a member of the SLC25 mitochondrial carrier family, the largest family of solute transporters in humans, the members of which share similar structural and mechanistic features (*6*, *7*). They transport a wide range of substrates, including nucleotides, amino acids, carboxylic acids, fatty acids, inorganic ions, and vitamins, thus linking the metabolic pathways of the mitochondrial matrix and cytosol and providing molecules and inorganic ions for maintenance and homeostasis (*6*, *7*).

The ADP/ATP carrier exists and functions as a monomer (*8*–*13*) and has a six-helical threefold pseudo-symmetric structure with a central substrate translocation pathway (*14*). Its structural fold consists of three domains (*15*), each containing two transmembrane helices that are linked by a loop and short matrix helix (*16*). The mitochondrial ADP/ATP carrier cycles between cytoplasmic-open and matrix-open states (*1*, *2*, *17*). The interconversion between the two states involves extensive synchronous movements of six structural elements: three core elements and three gate elements (*17*). In each of the three domains, the core element consists of the odd-numbered helix, matrix helix, and the N-terminal part of the even-numbered helix, whereas the gate element is the C-terminal part of the even-numbered helix (*17*). When the carrier cycles from the cytoplasmic-open state to the matrix-open state, the core elements rotate outward, opening the matrix side, while simultaneously the gate elements rotate inward, closing the cytoplasmic side of the carrier (*1*, *2*, *17*). These movements occur in reverse when the carrier converts from the matrix-open state to the cytoplasmic-open state (*1*, *2*, *17*). This transport mechanism is highly dynamic and unique to the members of the SLC25 family (*1*, *6*, *7*). To make the state interconversion possible, three cardiolipin molecules are tightly bound to the carrier, which link the domains together on the matrix side (*16*, *18*, *19*).

Three important functional elements are an integral part of this transport mechanism. The first, the matrix salt bridge network, forms when the carrier is in the cytoplasmic-open state and involves the charged residues of the [P]x[DE]xx[RK] motif, which are located on the core elements of helices H1, H3, and H5 (*18*). There is also a glutamine brace that interacts with the charged residues of a salt bridge, providing extra interactions to stabilize the network further (*16*).

The second functional element is a cytoplasmic salt bridge network that forms when the carrier is in the matrix-open state (*16*, *20*, *21*), which involves the charged residues of the [YF][DE]xx[RK] motif that are located on the gate elements of helices H2, H4, and H6 (*17*). This interaction network is strengthened by tyrosine braces, which consist of a hydrogen bond interaction between the hydroxyl group of the tyrosine residue of the motif with the negatively charged residue on the adjacent domain (*17*). Thus, the opening and closing of the carrier through coordinated movements of the six structural elements is accompanied by the disruption and formation of these two networks on either side of the carrier (*20*). In addition to the two networks, there are hydrophobic clusters or secondary structural elements that form ~15-Å insulation layers to aid the closure of the carrier as part of the state interconversion and to prevent the leak of molecules, protons, and other ions (*1*, *2*).

**Fig. 1.**
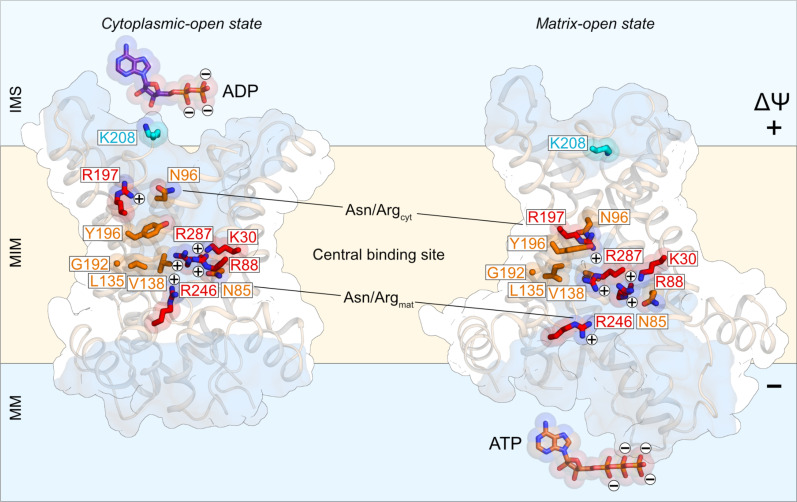
Positively charged residues putatively involved in charge movements. Lateral views of the cytoplasmic-open state [ScAac2, Protein Data Bank (PDB) code 4c9h, chain A] (**left**) and matrix-open state (TtAac, PDB code 6gci, chain A) (**right**) of the mitochondrial ADP/ATP carrier. The interacting residues of ScAac2 are conserved in TtAac, and to facilitate comparison, we used the TtAac labeling. The water-accessible surfaces, determined by HOLE59, are shown in transparent blue. The positively charged residues with an essential role in substrate binding are shown in red (red labeling), whereas neutral residues that are also important for substrate binding are shown in orange (orange labeling). K208, which serves as a control, is shown in cyan. The substrates ADP and ATP are shown in purple and salmon red, respectively. MM, mitochondrial matrix; MIM, mitochondrial inner membrane; IMS, mitochondrial intermembrane space.

The third functional element is a substrate-binding site (*20*, *22*, *23*), which allows the translocation of only ADP, ATP, and their deoxy variants (*24*–*26*). In contrast, adenosine 5′-monophosphate (AMP) can bind but is not transported (*24*–*26*) as it lacks the required interaction energy to initiate state interconversion (*23*, *27*). Recently, all residues involved in substrate binding were identified by studying the substrate-induced concentration-dependent thermostability shifts in single alanine replacement mutants (*28*), generating several important observations (Fig. 1). First, a central substrate-binding site was identified, which consists of three positively charged residues K30, R88, and R287, most likely involved in binding of the phosphate moieties, and residues L135, V138, G192, and Y196, most likely involved in binding of the adenosine moiety via hydrophobic and aromatic stacking interactions (Fig. 1). In addition, there are two pairs of asparagine/arginine residues: a N96/R197 pair on the cytoplasmic side (Asn/Arg_cyt_) and a N85/R246 pair on the matrix side (Asn/Arg_mat_), which are involved in substrate binding in a state-dependent manner (Fig. 1). These pairs are likely to be responsible for the initial binding and final release of the nucleotides while also controlling the geometry of nucleotide binding (*28*). All substrate-induced shifts are the same for ADP and ATP, indicating that the two substrates bind to the same set of residues with the same chemistry (*28*). Last, the transfer of substrates through the carrier is mirror symmetric, involving the two Asn/Arg pairs and the central site, explaining why ADP and ATP can be transported in either direction, fully reversibly.

All these functional and structural elements together support an alternating access mechanism in which a single substrate-binding site is accessible to one or the other side of the membrane. In agreement, it was recently demonstrated that the human mitochondrial ADP/ATP carrier has a ping-pong kinetic or double displacement mechanism, where one substrate is bound and transported, dissociating from the substrate-binding site before a second substrate is bound and transported in the opposite direction (*29*).

One of the key features of energy conversion in mitochondria is the generation of a proton motive force (*30*), which results from positive charge accumulating in the intermembrane space and negative charge in the mitochondrial matrix. This charge separation leads to the formation of an electric field in the membrane from the cytoplasmic side to the matrix side, resulting in a membrane potential of ~180 mV. The ping-pong kinetic mechanism of the mitochondrial ADP/ATP carrier (*29*) stipulates that ADP^3−^ (three negative charges) is imported against the membrane potential and ATP^4−^ (four negative charges) is exported with the membrane potential. In principle, moving the three negative charges on ADP^3−^ against 180 mV of the membrane potential represents a major potential barrier, which could prevent its import. The aim of this study is to understand the molecular mechanism by which the mitochondrial ADP/ATP carrier achieves this remarkable feat.

For this purpose, we expressed, purified, and reconstituted wild-type and mutant variants of the mitochondrial ADP/ATP carrier into liposomes and studied the charge movements induced by ADP or ATP binding and transport using solid-supported membrane-based electrophysiology (*31*, *32*). We show that positively charged binding site residues reorient with the negatively charged substrates from one compartment to the other. The total charge movement of these residues integrates to +3.3 per single transport step, explaining the observation that a net −0.7 charge is moved when ATP^4−^ is transported across and a net +0.3 charge is moved when ADP^3−^ is transported. Thus, our results explain why ADP^3−^ import and ATP^4−^ export are both stimulated and why ADP^3−^ export and ATP^4−^ import are both inhibited by the membrane potential in the context of a functional mitochondrion. In this way, the mitochondrial ADP/ATP carriers achieve high and directional adenine nucleotide exchange rates across the mitochondrial inner membrane to fuel the many metabolic energy-requiring processes of the eukaryotic cell.

## RESULTS

### Charge movements occur during adenine nucleotide transport by the ADP/ATP carrier

**Fig. 2.**
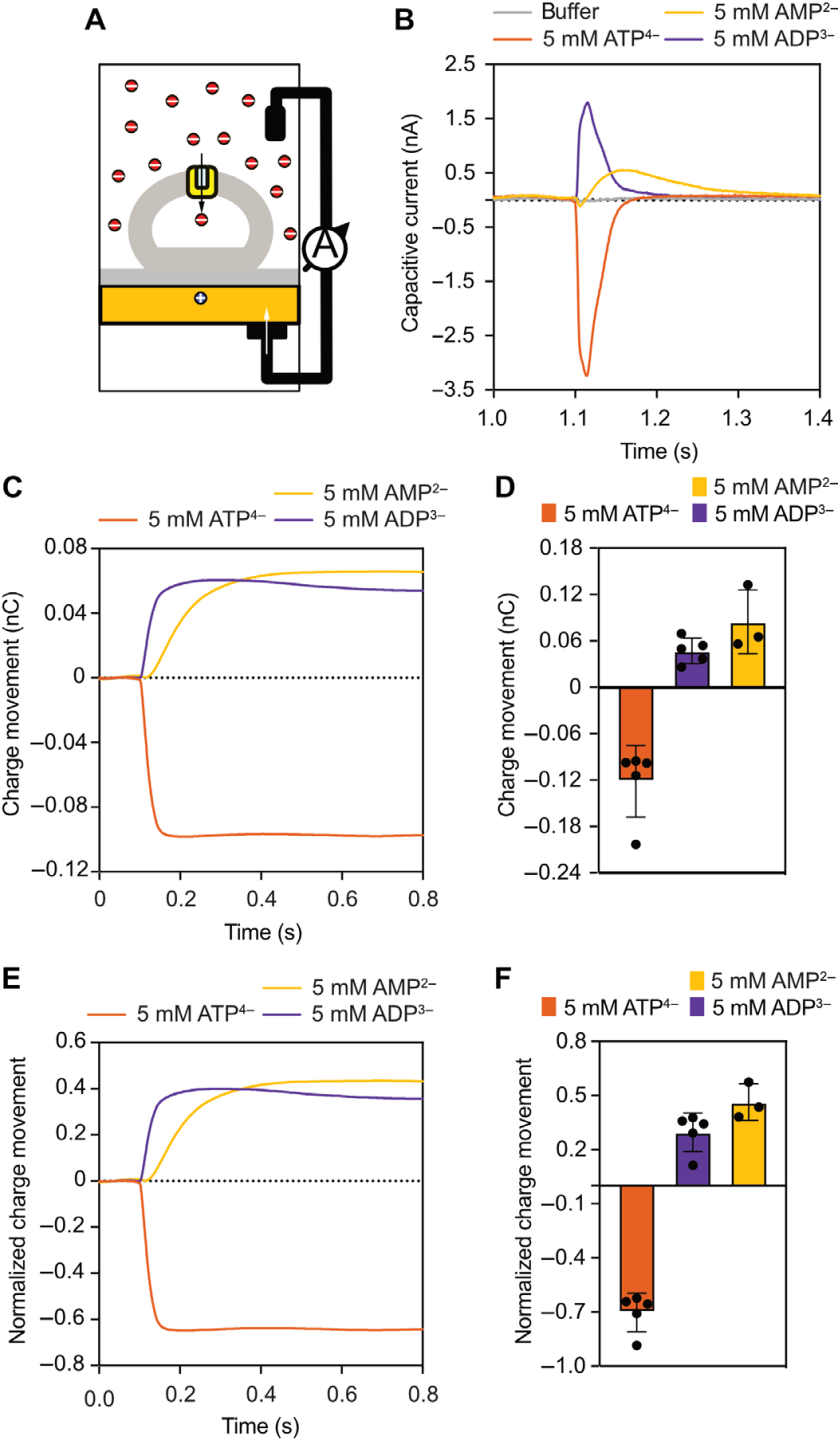
Charge movements by the mitochondrial ADP/ATP carrier. (**A**) Experimental setup for measurements of charge movements using solid-supported membrane-based electrophysiology. (**B**) Capacitive currents induced by 5 mM AMP, ADP, or ATP, measured by using empty liposomes (no internal substrate) in which the wild-type ADP/ATP carrier was reconstituted. The curves represent one example experiment, with each current being recorded twice. (**C**) Charge movements (nC) over time. Integrated data taken from (B). (**D**) Average of charge movements. The bars and error bars represent the mean and SD of three to five independent biological repeats. For each biological repeat, one to three sensor preparations were averaged, and each capacitive current was recorded twice on each sensor. (**E**) Normalized charge movements over time, using the formal charge difference between ADP and ATP. Data taken from (B). (**F**) Average of normalized charge movements, induced by 5 mM AMP, ADP, or ATP. The bars and error bars represent the mean and SD of three to five independent biological repeats.

To understand charge translocation during adenine nucleotide exchange, we reconstituted the wild-type mitochondrial ADP/ATP carrier into liposomes and measured the capacitive currents generated by ADP^3−^ or ATP^4−^ import into liposomes with no internal substrate by using solid-supported membrane-based electrophysiology (Fig. 2A). Charge separation as the result of charge movement into or across the membrane or protein dielectric generates an electric field that integrates over the width of the membrane to form a membrane potential. The magnitude of the membrane potential is proportional to the product of the charge and the dielectric distance that the charge moves, such that a single charge moving through half the membrane thickness would generate approximately half the membrane potential of a single charge moving across the complete membrane, when the membrane dielectric is homogeneous. The SURFE^2^R N1 measures a capacitive current, which is the product of the capacitance and the rate of change of the membrane potential. This current can be integrated over time to recover the charge movement, which is proportional to the membrane potential. There are two ways of generating a capacitive current: The first is the movement of a charge across the membrane dielectric, leading to the buildup of a membrane potential, and the second is the change of dielectric around a fixed charge. In the context of an alternating access mechanism, the first scenario could represent a transporter completing a transport step of a charged substrate, by closing one gate and by opening the other gate. The second scenario could represent a case where a charged substrate binds, inducing the closure of one gate but not the opening of the other gate, i.e., substrate binding without transport.

No capacitive current, and so no change in membrane potential, was observed during buffer exchange without nucleotides (Fig. 2B). In contrast, ADP^3−^ import generated a positive capacitive current, whereas ATP^4−^ import generated a negative capacitive current, both reflecting a single translocation step of those molecules into the liposomes per transporter on the sensor (Fig. 2B). The ADP/ATP carrier cannot change conformation in the absence of substrates due to the presence of the salt bridge networks (*1*, *2*). To exclude the possibility that these effects are due to nucleotide binding to liposomes, rather than carrier-mediated transport, we also prepared liposomes without protein and no charge movements were observed (fig. S1). Between subsequent measurements, a wash step was performed, so that the system returned to its initial state, and the substrates efflux from the liposomes (fig. S2). The measured capacitive current can be integrated to produce the charge movement (Fig. 2C), which can be averaged by using independent observations (Fig. 2D). Because the translocated ADP^3−^ and ATP^4−^ species differ by one formal charge, the difference in their charge movement represents one charge moving across the complete membrane, which can be used to normalize the charge movement of each substrate (Fig. 2E). In this way, it can be determined that, on average, an ADP^3−^ transport step leads to an overall charge movement of +0.3 ± 0.11, whereas an ATP^4−^ transport step leads to an overall charge movement of −0.7 ± 0.11 (Fig. 2F). These average values agree well with previous measurements carried out with liposomes containing reconstituted bovine ADP/ATP carrier absorbed onto black lipid membranes, giving +0.5 for the ADP^3−^ transport step and −0.5 for the ATP^4−^ transport step (*33*), as well as onto solid-supported membranes, giving +0.3 and −0.7, respectively (*34*). It is well established that only the unprotonated species (ATP^4−^ and ADP^3−^) are transported by the ADP/ATP carrier (*25*, *34*, *35*) and that there is no proton movement upon transport (*36*). Import of ADP or ATP moves −3 and −4 charges across the whole membrane, respectively. The observation that only +0.3 for ADP and −0.7 for ATP charges move across the membrane can best be explained by assuming that the carrier moves +3.3 charges across the membrane concomitantly. We also carried out these measurements in a range of substrate concentrations (fig. S3). As expected, there is a concentration-dependent signal amplitude (fig. S3A), but the charge transfer is the same in each concentration, as the +3.3 concomitant charge movement is an intrinsic property of the protein (fig. S3B).

We also measured the capacitive currents and charge movements after the addition of AMP^2−^, which binds to the carrier (*26*) but is not transported (*24*, *25*). The charge movement is positive, as expected, but relatively small compared to ADP^3−^-induced and ATP^4−^-induced currents (Fig. 2B). The integration and normalization show that AMP^2−^ binding generates a +0.46 ± 0.1 charge movement, which is much smaller than the expected +1.3 charge, had AMP^2−^ been transported (approximately one positive charge more than ADP^3−^). This observation is consistent with AMP^2−^ only moving across 33% of the membrane dielectric, rather than being fully translocated across the membrane. AMP^2−^ binding cannot provide enough interaction energy to overcome the interaction energy of the salt bridge networks and braces, which contain many polar and electrostatic interactions (*16*, *17*, *23*, *27*). Thus, AMP^2−^ is able to induce conformational changes that lead to partial closure of the cavity, not quite reaching the occluded state. These observations show that the capacitive current measurements can be used to characterize the binding of substrates to the carrier, as demonstrated recently for SGLT1 (*37*).

### Substrate-binding residues are potentially involved in positive charge transfer

Five positively charged residues have previously been shown to provide essential interactions between the substrates and the carrier (*28*). The most likely candidates for the charge transfer of +3.0 are the three positively charged substrate-binding residues K30, R88, and R287 that are present in the central substrate-binding site (Fig. 1). They are initially located inside a water-filled cavity exposed to one compartment, and they end up in a water-filled cavity exposed to the other compartment after substrate transport, i.e., they are fully translocated across the membrane dielectric. The remaining fractional value of +0.3 means that an integer charge has moved through only a fraction of the membrane dielectric. The other two charged residues, R197 and R246, are part of the Asn/Arg_cyt_ and Asn/Arg_mat_ pairs (Fig. 1) (*28*), and they only move partially through the membrane. In addition, a specific claim has been made for the involvement of the equivalent residue of K208 in substrate binding based on molecular dynamics simulations (*38*). However, this residue has been demonstrated to be involved in the state-specific formation of the cytoplasmic salt bridge network by two experimental methods (*17*, *21*). Nonetheless, we have included residue K208 in our analysis to evaluate its involvement in substrate binding and charge movements experimentally.

### Mutants of the five positively charged residues are unable to transport substrate

**Fig. 3.**
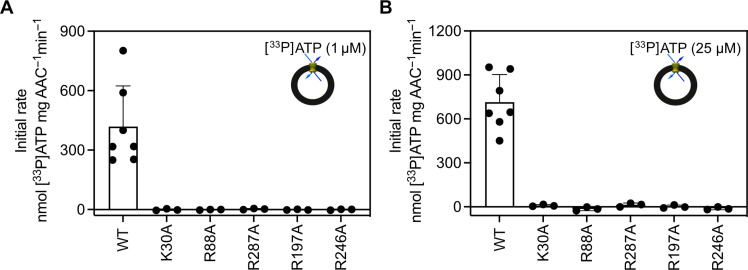
Alanine replacement mutants of the positively charged residues involved in substrate binding do not transport ATP. Initial transport rates determined from the linear part of the uptake curves. Proteoliposomes containing the wild-type (WT) or Ala variants were loaded with 1 mM unlabeled ATP. Transport was initiated by adding 1 μM (**A**) or 25 μM (**B**) [^33^P]ATP externally. Proteoliposomes with no internal substrate were used as controls, and the rates were subtracted from the rates achieved with loaded liposomes for each protein. The bars and error bars represent the mean and SD of seven biological repeats for the wild type and three for the variants. AAC, mitochondrial ADP/ATP carrier.

To determine if the aforementioned five positively charged residues are involved in charge movements, each residue was replaced by alanine (*28*). The isolated single alanine replacement mutants were pure and correctly folded, as evaluated by thermostability assays in the presence and absence of ligands (substrates and inhibitors) (*28*). Subsequently, the proteins were reconstituted into liposomes to assess their transport activities (Fig. 3). None of the five alanine mutants of the substrate-binding site showed substantial accumulation of substrate above background, showing that they were no longer capable of exchanging substrates. These results are in agreement with complementation assays, in which the same set of alanine replacement mutants were shown to be unable to support growth on the nonfermentable carbon source glycerol (*28*). Thus, all five positively charged residues have an essential role in the transport cycle, supporting a reversible mechanism.

### Positively charged residues of the central binding site are involved in charge transfer

The single replacement mutants of K30, R88, and R287, which are in the central substrate-binding site (Fig. 1), were subjected to capacitive current measurements using the same substrate concentrations and conditions as for the wild-type carrier (Fig. 2, B and C). These mutants are unable to transport (Fig. 3), and thus there is no current moving across the membrane. However, they still generated a capacitive current, implying that charge is being moved into the membrane dielectric, forming a membrane potential. Charge separation into a water-filled cavity is not expected to generate a membrane potential because of the high relative permittivity of water (ε_r_ ≈ 80) compared to the membrane (ε_r_ ≈ 4). Conversely, this means that the substrate binding into an open cavity is not sensitive to the membrane potential (*39*). Thus, to form a membrane potential and capacitive current, the substrate must bind, and the cavity must partially close toward the occluded state to draw the charge into the membrane, but not sufficiently to lead to its translocation, as was the case with AMP.

**Fig. 4.**
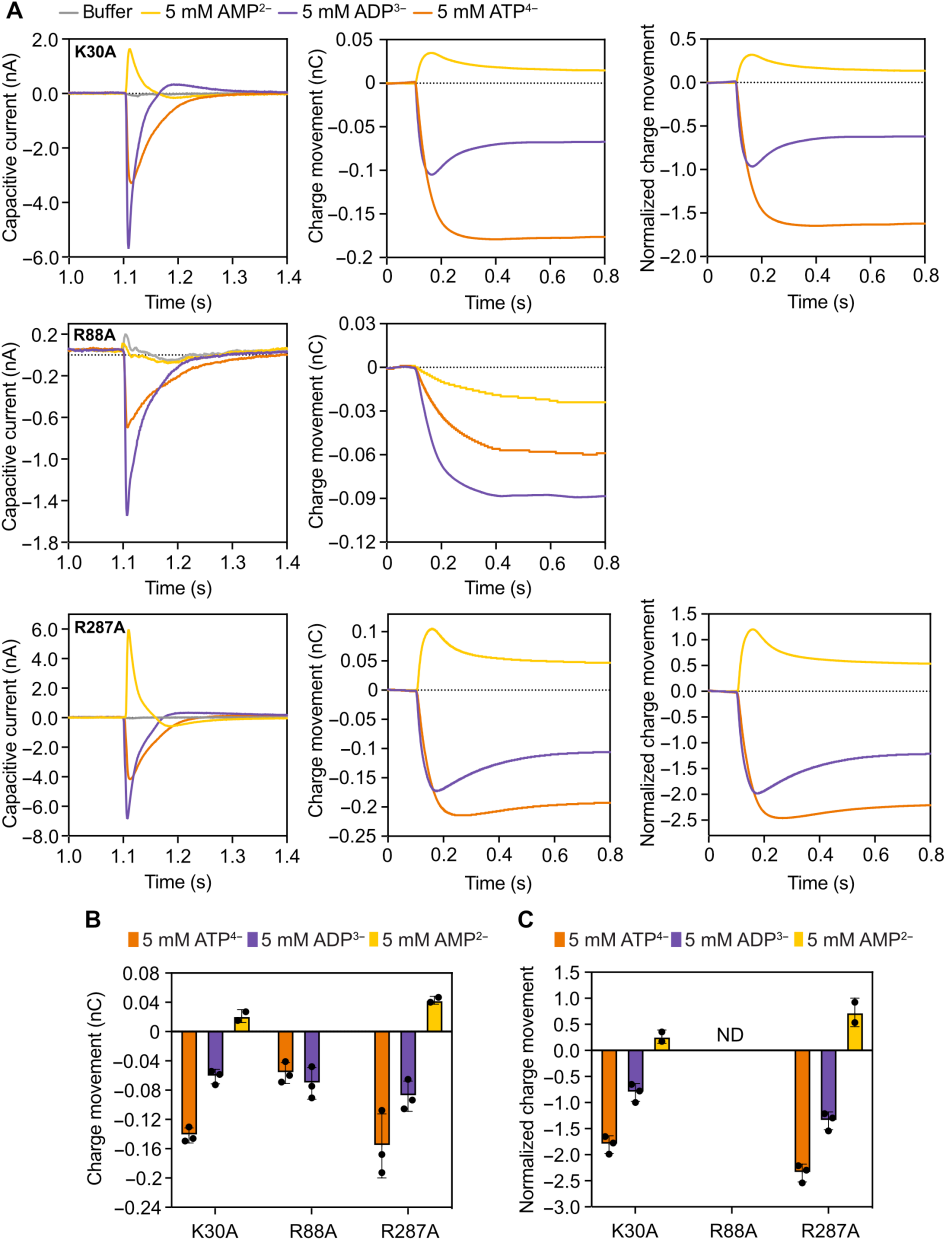
Charge movements by the central binding site replacement mutants of the mitochondrial ADP/ATP carrier. (**A**) Capacitive currents (nA), charge movements (nC), and normalized charge movements over time, induced by 5 mM AMP, ADP, or ATP for K30A (top), R88A (middle), and R287A (bottom). The capacitive currents were measured by using empty proteoliposomes (no internal substrate) in which the indicated mutant was reconstituted. The curves represent one example experiment, with each current being recorded twice. (**B**) Average of charge movements. The bars and error bars represent the mean and SD of two to three independent biological repeats. For each biological repeat, one to three sensor preparations were averaged, and each capacitive current was recorded twice on each sensor. (**C**) Averaged normalized charge movements over time by using the formal charge difference between ADP and ATP. The bars and error bars represent the mean and SD of two to three independent biological repeats. ND, not determined.

All three mutants generated a capacitive current upon the addition of adenine nucleotides and thus must bind an external substrate causing the cavity to close. However, the failure of the carrier to transport means that the cavity does not fully open to the inside of the proteoliposome to enable substrate release. All replacement mutants of K30, R88, and R287 show a negative charge movement for ATP^4−^, like the wild-type carrier, but in contrast to the wild type, ADP^3−^ also produces a negative charge movement (Fig. 4, A and B). The latter result proves that the substrate-binding site must contain less than three positive countercharges due to removal of one charge because of the mutation and that all three residues are involved in positive charge transfer during transport. As observed in the structures of the two conformational states (Fig. 1), these residues move fully from one compartment to the other together with the substrate; hence, they might contribute a full unit of charge each. In this case, the substrate-binding site of the mutants should contain +2.3 positive countercharges, resulting in a negative capacitive current for ADP^3−^ and naturally also for ATP^4−^.

The capacitive currents induced by ADP^3−^ and ATP^4−^ binding have different dynamics. ADP^3−^ binding–induced transitioning is fast, leading to a high amplitude in the capacitive current, and in some cases, that current is followed by a capacitive discharge (“overshoot”), typically observed with fast capacitive currents (*40*). In contrast, ATP^4−^ binding–induced transitioning is occurring more slowly and with a lower amplitude (Fig. 4A). The mutations have changed the number of the positive charges of the binding site compared to the wild-type carrier. The ADP^3−^ molecule is smaller, has fewer conformers, and might, upon binding, induce conformational changes faster than the ATP^4−^ molecule, which is larger, more negatively charged, and more conformationally diverse.

Although the alanine replacement mutants of K30, R88, and R287 are severely impaired in their function, we attempted to quantify the positive charges involved in their binding and transfer. If it is assumed that ATP and ADP binding result in the same degree of closure of the cavity and movement of the substrate and protein charge into the membrane, then the difference in the charge movement can be normalized to the formal charge difference to provide an estimate of the positive charges involved in the charge transfer. The best interpretable result was obtained with K30A, as substantially more negative charge is moved with the ATP^4−^ than with the ADP^3−^ binding–induced transitions. The analysis shows that the binding site contains +2.20 ± 0.21 charges, which is consistent with a mutated binding site that has lost a single positive charge. In turn, this result also means that the K30A mutant is still capable of conformational changes to bring out the formal charge difference between the two adenine nucleotides.

In the R88A mutant, more negative capacitive charge movement is induced by ADP^3−^ binding than by ATP^4−^ binding, which, at first glance, is an impossibility given the formal charge difference. However, this result means that the assumption of the same degree of closure is not valid and ADP^3−^ binding leads to a more substantive closure of the cavity than ATP^4−^ binding, which also agrees with the different closure dynamics induced by the binding of the two nucleotides. In the R287A mutant, more negative capacitive charge movement is induced by ATP^4−^ binding than by ADP^3−^ binding, but the difference accounts for a binding site with only +1.66 ± 0.17 positive charges, one formal charge less than expected. Therefore, the R287A might represent an intermediate case between K30A and R88A, where ADP^3−^ binding would still induce some closure, but less so in the case of ATP^4−^ binding, again reflecting the different closure dynamics induced by binding of the two. Both R88A and R287A mutants seem to be more impaired in the mechanism than K30A, which could relate to the fact these residues are contact points I and III of the substrate-binding site, respectively (*22*, *23*). These contact points are found in the substrate-binding sites of all mitochondrial carriers and have a key role in substrate recognition and binding (*22*, *23*), as well as in the conformational changes, as they form the hinge points between the core and gate elements (*17*). R88, which is contact point I, recognizes the type of substrate within a class, whereas R287 is contact point III, which is generically a positively charged residue (*22*, *23*). The important roles of these residues in both substrate binding and structural mechanism could explain why their contributions to the charge transfer could not be measured in contrast to that of K30.

For K30A and R287A, a positive current could be measured for AMP^2−^ (Fig. 4, A and B), in agreement with the notion that the remaining +2.3 charges of the mutated binding site would leave an overall positive charge upon binding of AMP^2−^. Addition of AMP^2−^ to the R88A mutant did not induce a capacitive current at all (Fig. 4A), indicating that this mutant is unable to generate any conformational changes upon addition of AMP, suggesting that R88 might be important for binding of the α-phosphate moiety. However, the highest charge movements were reproducibly measured for R287A, followed by K30A, whereas those recorded with R88A were lower (Fig. 4B). These differences might correlate to the structural stabilities of the mutant variants as the apparent melting temperatures of R287A, K30A, and R88A are 64.4° ± 0.2°, 57.3° ± 1.2°, and 53.1° ± 1°C, respectively, which are all higher than the melting temperature of the wild-type carrier at 50.2° ± 0.6°C (*28*). We have argued that the wild-type carrier is less stable than the mutants, i.e., at a higher energy level, because the three positive charges are located close together in the central cavity. Binding of the negatively charged adenine nucleotides to this site will lower the energy level by neutralizing the charges and by generating specific interactions, increasing the probability for conformational changes and substrate transport to occur (*27*). In the mutant variants, removal of one charge will not only stabilize the carrier more than the wild-type carrier, but will also affect adenine nucleotide binding, not generating sufficient energy input or the correct binding geometry required for the conformational changes, causing an impaired mechanism. The R287A mutant has the highest apparent melting temperature and thus is at the lowest energy level, which means that R287A might trigger conformation changes upon substrate binding more readily than R88A, explaining the higher currents.

### Two positively charged residues of the Asn/Arg pairs also make charge transfer contributions

**Fig. 5.**
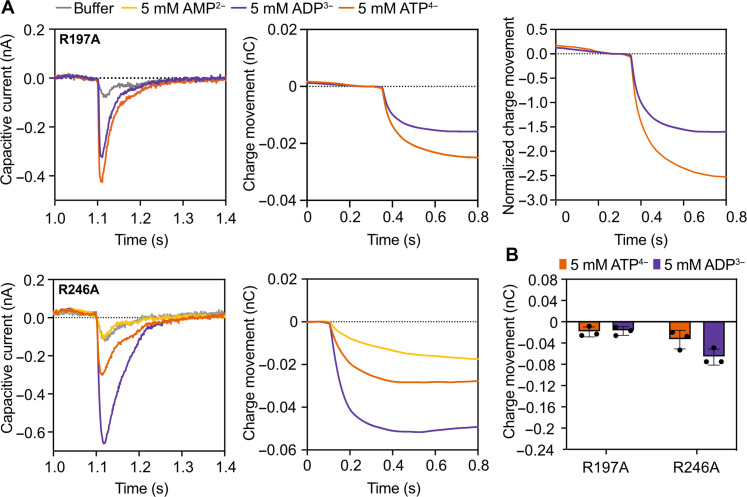
Charge movements by the replacement mutants of the Asn/Arg pairs of the mitochondrial ADP/ATP carrier. (**A**) Capacitive currents (nA), charge movements (nC), and normalized charge movements over time, induced by 5 mM AMP, ADP, or ATP for R197A (top) and R246A (bottom). The capacitive currents were measured by using empty proteoliposomes (no internal substrate) in which the indicated mutant was reconstituted. The curves represent one example experiment, with each current being recorded twice. (**B**) Average of charge movements. The bars and error bars represent the mean and SD of three independent biological repeats. For each biological repeat, one to three sensor preparations were averaged, and each capacitive current was recorded twice on each sensor.

The arginine residues of the Asn/Arg_cyt_ and Asn/Arg_mat_ pairs, R197 and R246, respectively, are also candidate residues for the positive charge transfer during transport (Fig. 1). The R197 and R246 mutants are unable to transport nucleotides (Fig. 3), but adenine nucleotide binding does generate small capacitive currents (Fig. 5), indicating that some closure of the carrier takes place in response to substrate binding. As observed for the mutants of the central binding site residues (Fig. 4), both ATP^4−^ and ADP^3−^ induce a negative capacitance current (Fig. 5A), demonstrating that these two Arg residues make contributions to positive charge transfer during transport. However, during the transport cycle, these residues move from the water phase to the protein phase and vice versa (Fig. 1), unlike the central binding site residues that fully move with the substrate from one water-filled compartment to the other. Therefore, it is likely that R197 and R246 only contribute a partial charge to the overall charge movements. Because a small negative current is generated for ADP^3−^, their charge contribution must be slightly more than +0.3, as the remaining positive charge of the binding site must be slightly smaller than the three negative charges of ADP^3−^. In R246A, ADP^3−^ binding induces more negative charge than ATP^4−^ binding, which must be due to ADP^3−^ binding generating a closure more readily. In R197A, the negative current is similar for ADP^3−^ and ATP^4−^, again not fully accounting for the formal charge difference, suggesting that ATP^4−^ binding–induced closure is relatively more affected. In conclusion, both residues make contributions to the positive charge transfer in each transport step as ADP^3−^ induces a negative current, but only a partial charge, slightly larger than +0.3.

### K208 is neither involved in substrate binding nor in positive charge transfer

On the basis of molecular dynamics simulations, it has been proposed that K208 is involved in substrate binding (*38*). However, the alanine replacement mutant of K208 is able to transport substrate and to support growth on glycerol (*21*), indicating that this residue is not essential for the overall transport mechanism. In addition, the purified mutant, after confirming its folded state and structural integrity (fig. S4, A and B), displays a stability shift in the presence of substrates, indicating that this residue is not critical for substrate binding (fig. S4C). Last, mutant K208A generates a positive current for ADP and a negative current for ATP (fig. S4D), like the wild-type carrier (Fig. 2B). Together, these results show that K208 is not involved in substrate binding or charge transfer, but they are consistent with K208 being a key residue of the cytoplasmic salt bridge network, in agreement with previous data (*16*, *17*, *21*).

## DISCUSSION

By studying the capacitive currents of single replacement mutants, induced by ADP^3−^ and ATP^4−^ binding, we have demonstrated that the central binding site residues K30, R88, and R287 and the arginine residues of the N96/R197 and N85/R246 pairs are all involved in the +3.3 charge transfer in each transport step (Figs. 1, 4, and 5). Thus, in the context of a functional mitochondrion, these residues reorientate with ADP^3−^ from the mitochondrial intermembrane space to the mitochondrial matrix during import and reorientate back with ATP^4−^ export, either fully or partially. The charge movements of the binding site residues are the same if the carrier is involved in import or export, in agreement with a fully reversible mechanism (Fig. 1), and thus the orientation of the reconstituted carriers does not affect the outcome of the experiments. It had been previously shown that single Ala mutations of the aforementioned residues abolish growth in complementation assays (*28*) and eliminate substrate-induced thermostability shifts (*28*). Here, we demonstrate that they are also involved in positive charge transfer with the substrate during translocation, providing independent evidence for their role in substrate binding and transport mechanism (Figs. 4 and 5).

These single alanine replacement mutants cannot complete a full transport cycle (Fig. 3), but they are still able to bind the substrate and generate a capacitive current through partial closure of the carrier to generate a membrane potential (Figs. 4 and 5). Similarly, AMP^2−^ binds to the wild-type carrier but is not transported, as it does not have sufficient binding interactions to overcome the energy barriers posed by the salt bridge networks (*27*). In all these cases, substrate binding leads to partial conformational changes toward the occluded state, but they are insufficient to lead to the complete set of conformational changes required for substrate translocation. Only in the case of K30A is there a sufficient difference between the capacitive currents with ADP^3−^ and ATP^4−^ to account for the difference of one formal charge. In this case, a charge transfer of +2.2 with the substrate agrees with the mutation removing one formal charge from the binding site. This result also shows that the transport mechanism of K30A is the least affected of all alanine replacement mutants, in agreement with this residue not being a contact point of the substrate-binding site (*17*, *22*, *23*). In the case of the other mutants, there are variable degrees of impairment of the mechanism induced by substrate binding, most often in response to ATP^4−^ binding (Figs. 4 and 5). Both nucleotides induce similar thermostability shifts for the wild-type and alanine replacement mutants within experimental error (*28*), indicating that all of the interactions ADP and ATP have with the substrate-binding residues are the same. Yet, ADP^3−^ binding to the central site mutants induces faster conformational changes than ATP^4−^ binding (Fig. 4), which must be due to differences in the properties of the two substrates. ADP^3−^ is smaller, has less negative charge, and has less conformers than ATP^4−^, suggesting that it can adapt to the mutated site more readily to induce transitions. The dynamics are the same for both nucleotides in the wild-type carrier, indicating that the binding site has evolved to bind both substrates with similar efficacies to trigger conformational changes (*28*).

In the wild-type carrier, a single transport step of ADP^3−^ moves a +0.3 charge and a single transport step of ATP^4−^ moves a −0.7 charge overall, which agree well with observations made for the bovine carrier (*33*, *34*). The most straightforward explanation is that +3.3 charges move with the substrates from one compartment to the other. In this work, we demonstrate the direct involvement of the central substrate-binding site residues K30, R88, and R287 and the N96/R197 and N85/R246 pairs (Figs. 4 and 5) in the positive charge transfer. All single replacement mutations of these residues change the positive capacitive currents induced by ADP^3−^ in the wild type to negative currents, providing proof in principle of their involvement. The control K208A gave a positive capacitive current with ADP^3−^, demonstrating that it is not involved in charge transfer during transport, in agreement with thermostability, complementation, and transport assays (fig. S4) (*28*).

The overall charge movement of +3.3 is an integration of all charge movements that occur within the carrier when the substrate is transported, but by combining them with structural information, we can tentatively assign them. The three central site residues move together with the substrate from the water-filled cavity in one conformational state to the water-filled cavity in the other state via an occluded state (Fig. 1). On this basis, it is likely that each contributes one formal charge and jointly approximately +3 of the +3.3 total charges. This estimate is reasonable as K30A and R287A replacement mutants show a negative current for ADP^3−^ (+2.3 − 3.0 = −0.7) and a positive current for AMP^2−^ (+2.3 − 2.0 = +0.3).

The Arg/Asn pairs are in the matrix and cytoplasmic gates of the carrier, which are crucial to opening and closing of the carrier from one side of the membrane to the other in an alternating way. These positions mean that the Arg/Asn pairs move quickly from the water phase to a proteinaceous environment and vice versa, possibly integrating to a partial charge movement of about +0.3 in each transport step. Because the capacitive currents of the Asn/Arg replacement mutants are consistently small, but still negative for ADP^3−^, their contribution to the overall charge movements must therefore be slightly larger than +0.3.

**Fig. 6.**
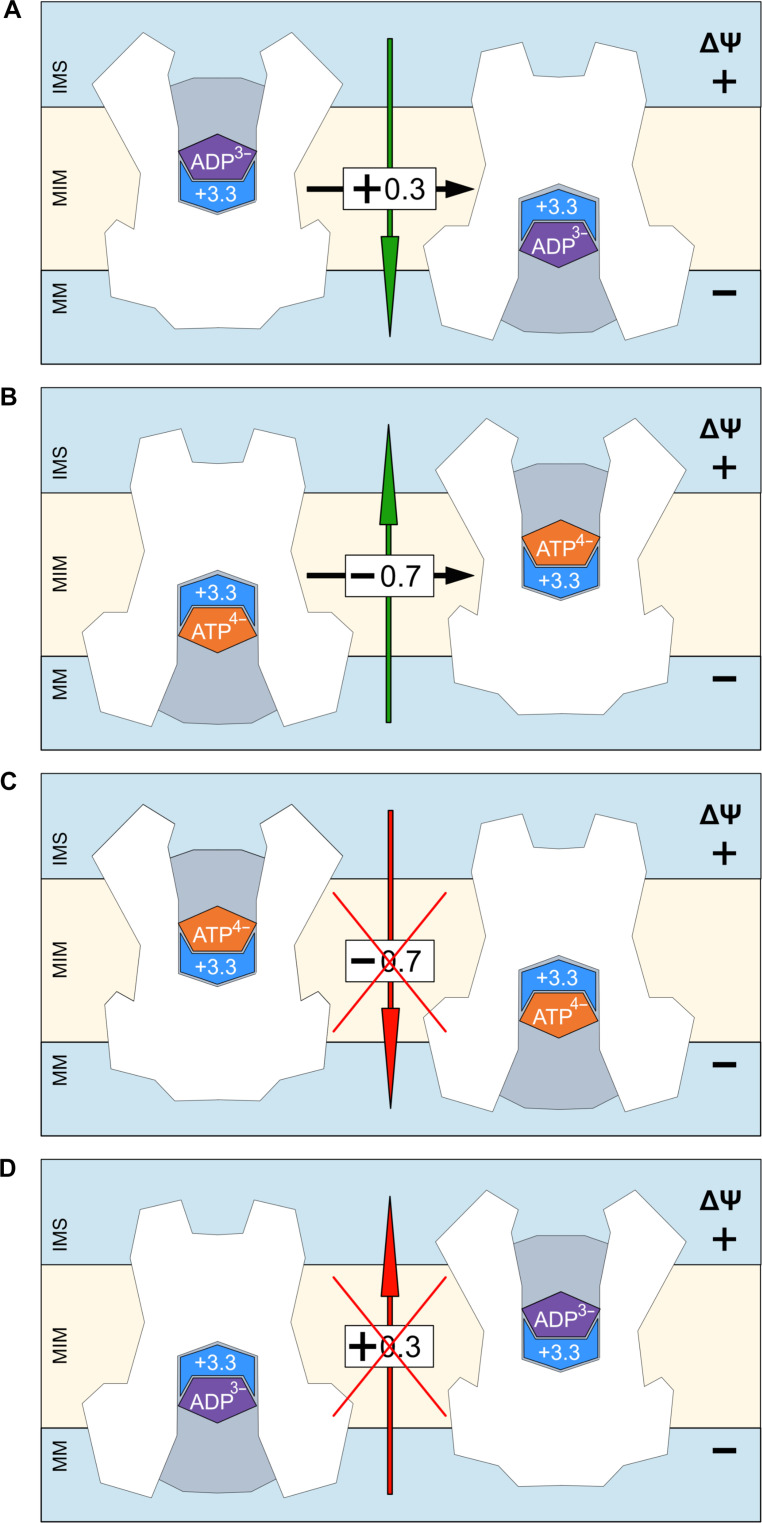
Charge movements dictate the directionality of adenine nucleotide transport in the presence of a membrane potential. (**A**) Import of ADP and (**B**) export of ATP, transport steps that are both stimulated by the membrane potential. (**C**) Import of ATP and (**D**) export of ADP, transport steps that are both opposed by the membrane potential because of charge movements of the substrate-binding site residues. The substrates ADP and ATP are shown in purple and salmon red, respectively, whereas the +3.3 charges of the binding site of the carrier are shown schematically in blue.

These positive charge transfers within the carrier with the transported adenine nucleotide are crucial for its operation in the context of a functional mitochondrion. When ATP^4−^ is exported, a −0.7 charge is overall moved along the electric field of the membrane potential (positive outside), stimulating this transport step. More notably, the membrane potential also stimulates the import of ADP^3−^ as, overall, a net +0.3 charge is also moved along the electric field of the membrane potential (negative inside) (Fig. 6). Asn/Arg pairs help nucleotides in and out of the central binding site in an ordered manner, allowing a fully reversible mechanism (*28*). Here, we show that they have also evolved to allow both the ADP^3−^ import step and ATP^4−^ export step to be driven by the membrane potential to achieve the high directional fluxes across the inner membrane, to fuel the metabolic energy-requiring processes of the cell.

Typically, ATP concentrations are kept high and ADP concentrations are kept low in the cytosol, to drive ATP hydrolysis used for metabolic energy-requiring reactions and processes by maintenance of a mass-action ratio. Because the intermembrane space is confluent with the cytosol through voltage-dependent anion channels and because there is only one binding site in the mitochondrial ADP/ATP carrier, ATP could, in principle, compete with ADP for import at the binding site. It should be noted that enzymes located nearby use ATP, such as hexokinase and creatine kinase, to generate ADP to maintain substrate concentration gradients across the inner membrane, but that is not the complete picture. As shown here, ATP import would lead to an overall charge transfer of −0.7 against the membrane potential and thus would be strongly opposed (Fig. 6). Similarly, phosphate and ADP concentrations in the mitochondrial matrix need to be kept high to drive the synthesis of ATP, meaning that ADP could, in principle, compete with the synthesized ATP for export. Because a net +0.3 charge is transported with ADP, the export step would also be inhibited by the membrane potential (Fig. 6). The mitochondrial ADP/ATP is a fully reversible molecular machine, which operates in a thermal bath (*27*), meaning that every step of the transport cycle is fully reversible. The combination of the membrane potential and the transported charge (substrate charge plus transporter charge) affects the forward and reverse rate constants of each step. The effect of the membrane potential is to apply a small Newtonian force on the substrate that pushes ADP toward the matrix side and pushes ATP toward the cytosolic side. The thermal forces are random in direction, averaging to zero, such that the membrane potential biases the transporter to import ADP and export ATP. Thus, the net result is that ATP is always reversed out whereas ADP moves toward the matrix. Similarly, ADP is reversed inward, whereas ATP moves toward the intermembrane space. Thus, the membrane potential is crucial for the maintenance of high directional adenine nucleotide exchanges across the inner membrane, by stimulating the import of ADP and export of ATP while inhibiting the export of ADP and import of ATP by the carrier.

What would happen if there was a collapse of the membrane potential in mitochondria, for example, under conditions where the electron transfer chain would fail due to loss of mitochondrial DNA (ρ0 mutants) or lack of oxygen? Transport of adenine nucleotides would then be driven by their concentration gradients because both nucleotides can be transported in either direction in the absence of a membrane potential. ATP, synthesized in the cytosol by glycolysis, would be imported by the mitochondrial ADP/ATP carrier down its concentration gradient. Each time an ATP molecule is imported, a net −0.7 charge is moved inside the mitochondrion, contributing to the buildup of a membrane potential. The imported ATP could then be used for ATP-requiring maintenance processes. ADP generated by hydrolysis would be exported, also contributing to the buildup of a membrane potential by the movement of a +0.3 charge out of the mitochondrion. Thus, in this way, the mitochondrial ADP/ATP carrier would be instrumental to the maintenance of a membrane potential, stimulating mitochondrial protein import and molecule transport, and would also provide glycolytic ATP for energy-requiring mitochondrial functions, such as mitochondrial DNA replication and protein synthesis.

A systematic analysis of the properties of the substrate-binding sites of mitochondrial carriers of the SLC25 family (*1*, *6*, *7*, *41*) has shown that charge transfer with the substrate might be a key feature of all of them (*20*, *22*, *23*, *42*). Because many of them are anion exchangers, positively charged residues in the substrate-binding site facilitate substrate import into mitochondria against the membrane potential, as observed here for the mitochondrial ADP/ATP carrier. Some mitochondrial carriers, such as the mitochondrial phosphate carrier, citrate carrier, and aspartate/glutamate carrier (*1*, *6*, *7*), also have negatively charged residues in the binding site, which could be involved in proton coupling (*43*). The purpose of proton symport is not to facilitate the import of anions against the membrane potential per se, but to drive the uptake of the molecules against their concentration gradient to maintain high pools in the mitochondrial matrix (*43*). Further analysis of these critical residues involved in substrate binding and proton coupling is required to understand these principles fully.

## MATERIALS AND METHODS

### Construction of expression strains

The mitochondrial ADP/ATP carrier of *Thermothelomyces thermophila* was the subject of the study. For expression of the wild-type carrier, the gene was codon optimized and cloned in a pYES3/CT derivative vector (Invitrogen), containing the constitutively active pPIC2 promoter of the phosphate carrier *pic2* of *Saccharomyces cerevisiae* (*8*, *21*, *28*). The single alanine replacements have been described previously (*28*). The expression plasmids were transformed into the *S. cerevisiae* strains WB-12 (MATα *ade2-1 trp1-1 ura3-1 can1-100 aac1::LEU2 aac2::HIS3*) (*44*), in which *aac1* and *aac2* genes are disrupted, or in strain W303-1B (MATα *leu2-3, 112 trp1-1 can1-100 ura3-1 ade2-1 his3-11,15*), using the lithium acetate/single stranded carrier DNA/ polyethylene glycol method (*45*). Successful transformants were selected on synthetic-complete tryptophan-dropout medium (Formedium) plates, supplemented with 2% (w/v) glucose.

### Large-scale expression and mitochondrial preparations

For each wild-type and variant protein, a 1-liter preculture was used to inoculate 10 liters of YPG medium plus 0.1% glucose in an Applikon autoclavable 15L Bio-bundle with eZ control. For expression of the wild-type carrier, WB-12 was used as the genetic background, whereas for variants, the W303-1B strain was used as this strain allowed for sufficient expression, irrespective of the functional state of the mutant protein. Precultures were grown in the selection medium [synthetic-complete tryptophan-dropout medium, supplemented with 2% (w/v) glucose] and were used to inoculate the 10-liter YPG medium containing 0.1% (w/v) glucose, in an Applikon Pilot Plant 140-l bioreactor (*46*). Cells were lysed using a DYNO-MILL (Willy A. Bachofen), and mitochondria were isolated using established procedures (*46*), snap frozen in liquid nitrogen, and stored at −70°C until further use.

### Protein purification

To purify the protein, isolated yeast mitochondria (~350 mg of total protein) were solubilized by resuspension in a buffer containing 20 mM tris-HCl (pH 7.5), 10% (v/v) glycerol, 150 mM NaCl, 20 mM imidazole (pH 7.5), one cOmplete EDTA-free protease inhibitor cocktail tablet (Roche), and 1% (w/v) dodecyl-β-maltoside (Glycon Biochemicals GmbH) followed by incubation under gentle agitation at 4°C for 1 hour. The particulate material was removed by ultracentrifugation (235,000*g*, 45 min, 4°C), and the soluble fraction was incubated with previously washed and equilibrated [20 mM tris-HCl (pH 7.5) and 150 mM NaCl) nickel Sepharose beads (GE Healthcare) at 4°C for 2 hours, under gentle agitation. Unbound proteins were removed by centrifugation (200*g*, 4°C, 5 min), and the bound fraction was placed in an empty column (Bio-Rad), where it was washed with 40 column volumes of Buffer A [20 mM Hepes-NaOH (pH 7.5), 150 mM NaCl, 20 mM imidazole (pH 7.5), 0.1% (w/v) dodecyl-β-maltoside, and tetraoleoyl cardiolipin (0.1 mg/ml)], followed by 25 column volumes of Buffer B [20 mM Hepes-NaOH (pH 7.5), 150 mM NaCl, 0.1% (w/v) dodecyl-β-maltoside, and tetraoleoyl cardiolipin (0.1 mg/ml)]. The column material was resuspended with 500 μl of Buffer B and supplemented with 5 mM CaCl_2_ and 10 μg of Factor Xa protease (New England Biolabs) for on-column digestion overnight at 10°C, with gentle agitation. For variants R197A and R246A, the on-column digestion step was reduced to 2 hours, using 30 μg of Factor Xa protease, due to protein instability over time. Following Factor Xa treatment, the cleaved protein was separated from the nickel Sepharose resin with an empty Proteus Midi spin column (Generon) (200*g*, 5 min, 4°C). Protein concentrations were measured with a spectrophotometer (NanoDrop Technologies) at 280 nm.

### CPM thermostability shift assays

Thermal stability assays in the presence and absence of effectors (substrates or inhibitors) were performed using a temperature ramp, as previously described (*47*). The temperature increase leads to initially inaccessible cysteine residues becoming exposed, due to protein unfolding, and reacting with the fluorophore *N*-[4-(7-diethylamino-4-methyl-3-coumarinyl)phenyl]maleimide (CPM) (*48*) to form fluorescent adducts. Fluorescence is monitored by using a rotary quantitative polymerase chain reaction (qPCR) instrument (Rotor-Gene Q, Qiagen). For each experiment, a CPM stock solution (5 mg/ml) in dimethyl sulfoxide was diluted 50-fold in purification Buffer B [20 mM Hepes-NaOH (pH 7.5), 150 mM NaCl, 0.1% (w/v) dodecyl-β-maltoside, and tetraoleoyl cardiolipin (0.1 mg/ml)] and was equilibrated in the dark for 10 min at room temperature. Three micrograms of the purified protein was mixed with the relevant effector (where indicated) and diluted into Buffer B to a final volume of 45 μl, to which 5 μl of the CPM solution (0.1 mg/ml) was added. The final concentration of each effector is indicated in the figure. The mixture was vortexed briefly and incubated in the dark for 10 min at 4°C, before being placed in the qPCR instrument. Subsequently, the protein population was assayed in a temperature gradient from 25° to 90°C, corresponding to a rate of ~4°C/min. The increasing fluorescence emitted by the protein-fluorophore adduct was measured in the High Resolution Melt channel of the machine (excitation at 440 to 480 nm and emission at 505 to 515 nm). Unfolding profiles were analyzed with the Rotor-Gene Q software 2.3, where the inflection point of the unfolding curves was used to determine the apparent melting temperature (*T*_m_). A temperature shift (Δ*T*_m_) is the difference between the *T*_m_ in the presence and absence of effectors.

### Protein reconstitution for transport studies

Egg l-α-phosphatidylcholine and tetraoleoyl cardiolipin (18:1) were mixed in a 20:1 (w/w) ratio and dried under a stream of nitrogen. Dried lipids were kept on ice. The lipids were rehydrated (final concentration of 10 mg ml^−1^) in a buffer containing 20 mM Hepes-NaOH (pH 7.5), 50 mM NaCl, and, when indicated, 1 mM ATP. Solubilization was performed with pentaethylene glycol monodecyl ether (C_10_E_5_) at a final concentration of 1.5% (v/v) by vortexing. Approximately 15 μg (for the wild type) or 20 μg (for the variants) of the purified protein was added to the mixture (lipid-to-protein ratio of 600 to 800:1 w/w) and incubated for 5 min on ice. Liposomes were formed by stepwise removal of the C_10_E_5_ using additions of SM-2 bio-beads (Bio-Rad). Four additions of 60 mg followed by one addition of 480 mg were made every 20 min, while the samples were inverting at 4°C. The samples were incubated overnight at 4°C with inversion. Bio-beads were removed the next day by passage through empty micro-bio spin columns (Bio-Rad). The external substrate was removed by gel filtration, shortly before the initiation of the transport reaction, using a PD10 desalting column (GE Healthcare).

### Transport assays

Transport assays were carried out with a Hamilton MicroLab Star robot (Hamilton Robotics). One hundred microliters of proteoliposomes in a 20 mM Hepes-NaOH (pH 7.5) and 50 mM NaCl buffer was loaded per well in a MultiScreenHTS-HA 96-well filter plate (pore size = 0.45 μm; Millipore). Uptake of radiolabeled [^33^P]ATP (Hartmann Analytic) in exchange for unlabeled ATP was initiated by the addition of 1 or 25 μΜ [^33^P]ATP. The uptake of [^33^P]ATP was stopped after 0, 10, 20, 30, 45, 60, and 150 s and 5, 7.5, and 10 min by addition of 200 μl of an ice-cold Hepes-NaOH buffer (pH 7.5) and 50 mM NaCl, and the samples were filtered with a vacuum manifold, followed by two additional wash steps with 200 μl of an ice-cold buffer. Plates were dried overnight, and the radioactivity was measured by adding 200 μl of MicroScint-20 (PerkinElmer) and by using a TopCount scintillation counter (PerkinElmer).

### Protein reconstitution into liposomes for solid-supported membrane electrophysiology

Egg l-α-phosphatidylcholine and tetraoleoyl cardiolipin (18:1) were mixed in a 20:1 (w/w) ratio and dried under a stream of nitrogen. The lipids were rehydrated in a final concentration of 5 mg ml^−1^ in a buffer containing 20 mM Hepes-NaOH (pH 7.5) and 50 mM NaCl. Solubilization was performed with C_10_E_5_ at a final concentration of 0.25% (v/v) by vortexing. Purified proteins, concentrated to 2 to 3 mg ml^−1^ (using a 50-kDa molecular weight cut-off concentrator) were added at a lipid-to-protein ratio of 25:1 (w/w) and incubated for 5 min on ice. Liposomes were formed by stepwise removal of the C_10_E_5_ using additions of SM-2 bio-beads (Bio-Rad, United Kingdom). Five additions of 20 mg followed by one addition of 160 mg were made every 20 min, while the samples were inverting at 4°C. The samples were incubated overnight at 4°C with inversion. Bio-beads were removed the next day by passage through empty micro-bio spin columns (Bio-Rad, United Kingdom), and the samples were frozen in liquid nitrogen.

### Sensor preparation

Capacitive currents were recorded by using new 3-mm diameter sensors (Nanion Technologies GmbH). Sensors were prepared as previously described (*31*). First, 50 μl of 0.5 mM octadecanethiol (Sigma-Aldrich, O1858) in isopropanol (Sigma-Aldrich, W292907) was added to the sensor and incubated for 30 to 60 min at room temperature in a closed petri dish. Subsequently, the sensors were rinsed three times with isopropanol and three times with deionized water and dried thoroughly by tapping on tissue paper. Two microliters of 1,2-diphytanoyl-*sn*-glycero-3-phosphocholine (7.5 mg/ml; Avanti Polar Lipids Inc., 850356, 25 mg of powder) in *n*-decane (Sigma-Aldrich, 8.03405 EMD Millipore) was pipetted on the sensor surface, followed by immediate addition of 50 μl of a nonactivating solution [30 mM Hepes-NaOH (pH 7.4) and 140 mM NaCl] to spontaneously form the solid-supported membrane. The sample (proteoliposomes or liposomes for the control) was diluted 1:5 in a nonactivating solution and sonicated in a bath sonicator (three cycles of 10 s each, with 20-s recovery time between them). Ten microliters of the sonicated sample was added to the sensor by submerging the pipette tip halfway in the solution, covering the sensor and dispensing very slowly. Subsequently, sensors were centrifuged (2000*g*, 15°C, 30 min) and stored at 4°C until used for the experiment (max a few hours). The sensor quality was evaluated by measurements of capacitance and conductance using the SURFE^2^R N1 Control software (V1.6.0.1). Only sensors with a capacitance of 1 to 40 nF and a conductance of <12 nS were used for measurements.

### Solid-supported membrane-based electrophysiology measurements

The electrophysiological measurements were performed with the SURFE^2^R N1 instrument (Nanion Technologies), as described in (*31*). A single solution exchange program was used, which consisted of three phases of 1 s each. A nonactivating buffer [30 mM Hepes-NaOH (pH 7.4) and 140 mM NaCl) or an activating buffer [30 mM Hepes-NaOH (pH 7.4) and 140 mM NaCl, containing a nucleotide at the indicated concentration] was sequentially injected into the sensor at a flow rate of 200 μl/s. Only the currents upon addition of the activating solution are shown and used for analysis. The measurements were repeated two to three times on each sensor. The presented data are the average and SD of two to four independent biological repeats (different purifications and reconstitutions), each containing the average of one to three sensors on which each current was recorded twice. The capacitive current was integrated to a charge movement in nanocoulombs by first offsetting the currents to zero at long time points to a mean of zero. The trace was then integrated with a simple trapezium algorithm, and then the baseline data immediately prior to addition of a substrate was offset to a mean of zero. This charge movement was then normalized so that the difference between ADP^3−^ and ATP^4−^ at long time points was 1.0 to produce the charge moved during translocation.
