# Supplementary material for: Membrane potential stimulates ADP import and ATP export by the mitochondrial ADP/ATP carrier due to its positively charged binding site
Source: Sci Adv. 2024 Nov 1;10(44):eadp7725. doi: 10.1126/sciadv.adp7725 (PMC11529707; doi:10.1126/sciadv.adp7725)
Supplement: Supplementary file 1 — Figs. S1 to S4 [file sciadv.adp7725_sm.pdf]

Science Advances
AAAS

Supplementary Materials for

Membrane potential stimulates ADP import and ATP export by the mitochondrial ADP/ATP carrier due to its positively charged binding site

Vasiliki Mavridou *et al.*

Corresponding author: Edmund R. S. Kunji, ek@mrc-mbu.cam.ac.uk

Sci. Adv. **10**, eadp7725 (2024)
DOI: 10.1126/sciadv.adp7725

This PDF file includes:

Figs. S1 to S4

Supplementary figures

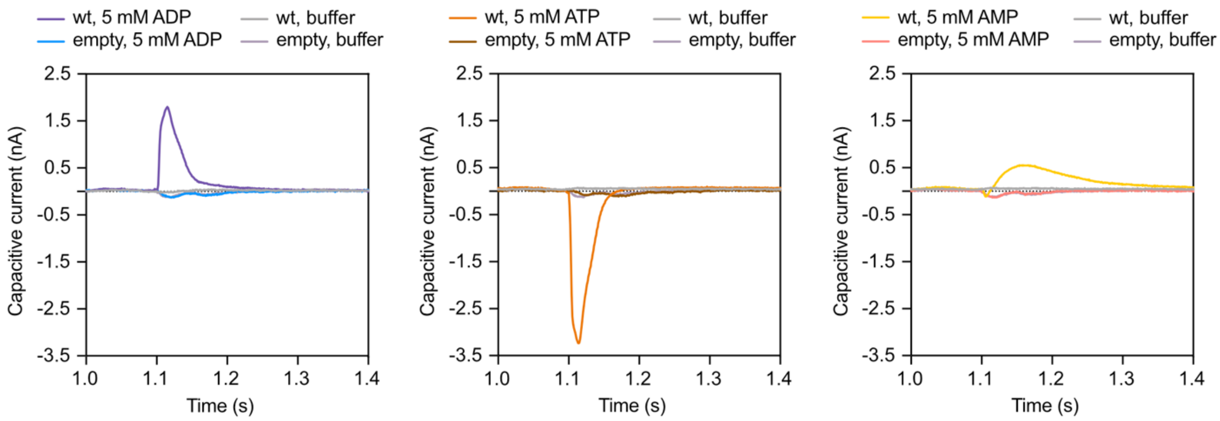

Fig. S1: Background binding evaluation using empty liposomes. Capacitive currents induced by buffer or 5 mM ADP (left), ATP (middle) and AMP (right) were measured using unloaded liposomes with no protein reconstituted and for comparison with the wild-type protein reconstituted. The curves represent one example experiment. Each current was recorded twice and averaged.

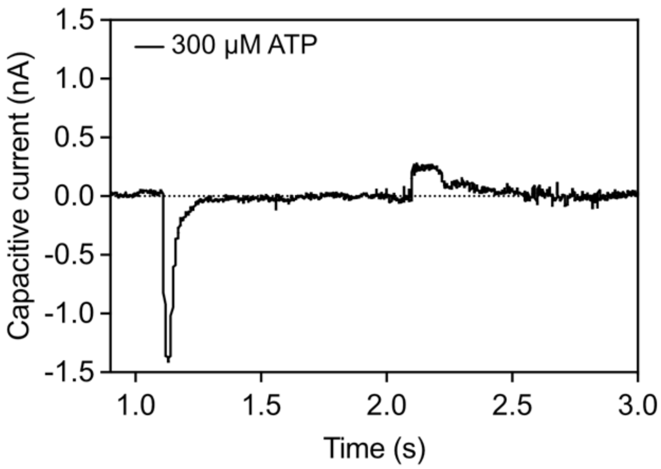

Fig. S2. Example trace showing the experimental set-up. A single solution exchange program was used. At 1.0 s activating buffer (30 mM HEPES-NaOH pH 7.4, 140 mM NaCl, containing nucleotide at the indicated concentration, here 300 μM ATP) was injected on the sensor, followed at 2.0 s by non-activating buffer (30 mM HEPES-NaOH pH 7.4, 140 mM NaCl). In the last phase (non-activating buffer) the substrate slowly effluxes from the proteoliposomes.

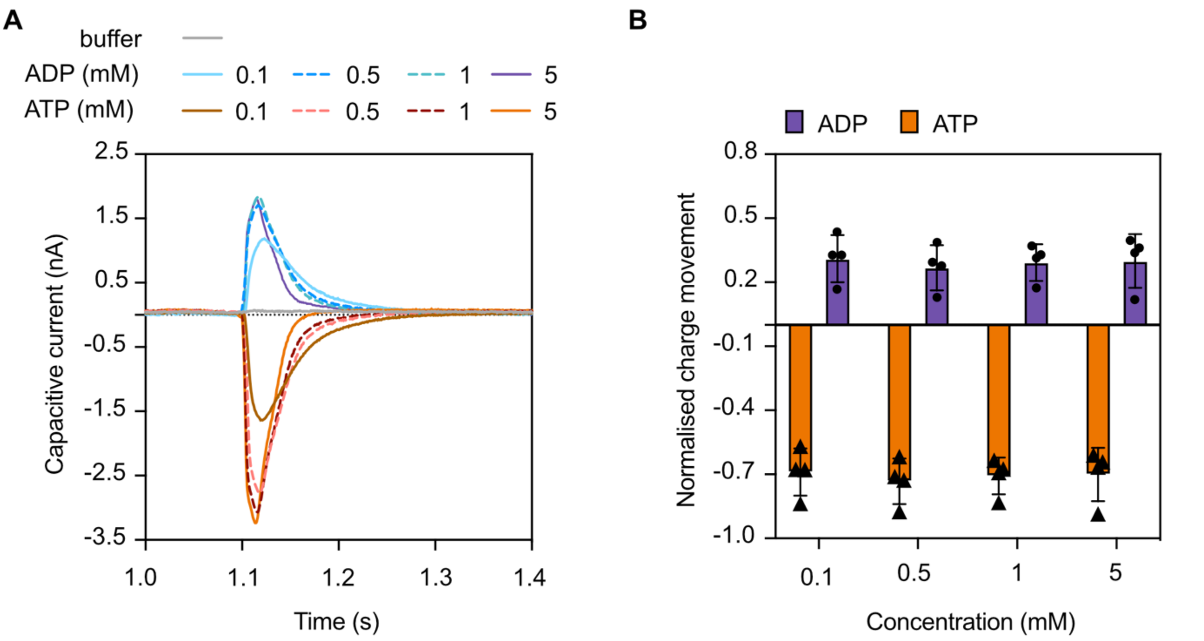

Fig. S3. Charge transfer by the mitochondrial ADP/ATP carrier is not concentration dependent. (A) Capacitive currents induced by 0.1, 0.5, 1, and 5 mM ADP or ATP, measured as described in Fig 2A. The curves represent one example experiment, with each current being recorded three times. **(B)** Average of normalized charge movements, induced by 0.1, 0.5, 1, and 5 mM ADP or ATP using the formal charge difference between ADP and ATP, as described in Fig 2E. The bars and error bars represent the mean and SD of 4 independent biological repeats.

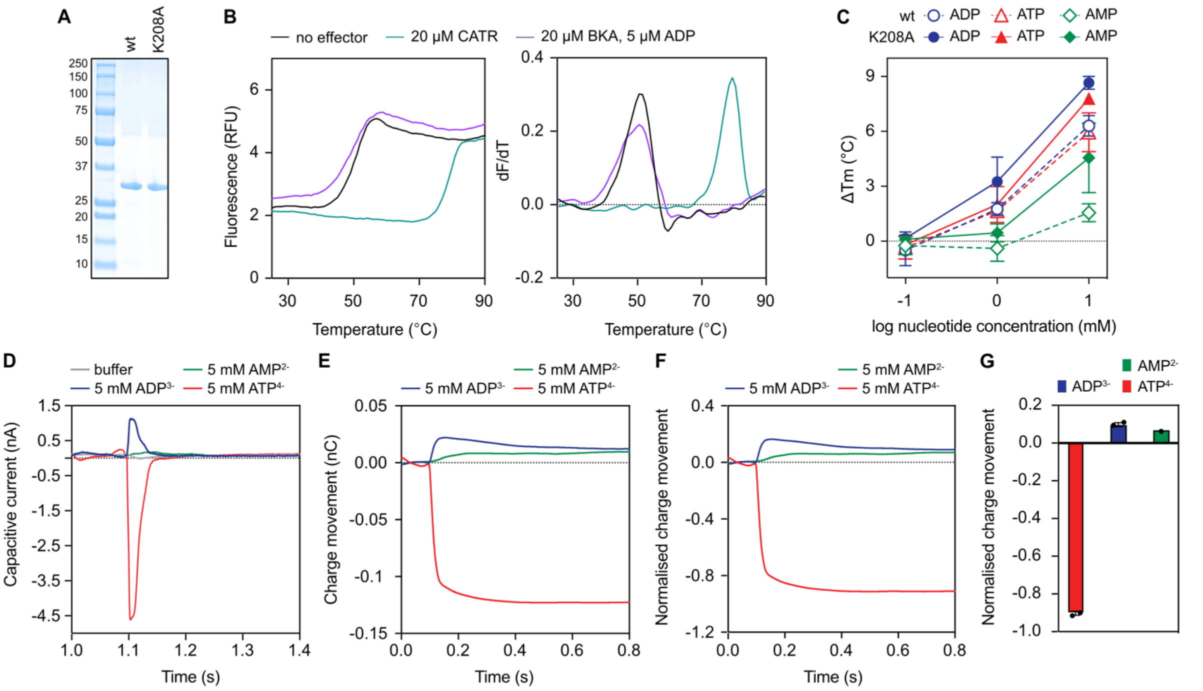

Fig. S4. K208 is not involved in substrate binding or positive charge transfer.

Purification of the wild type and K208A mutant protein. **(A)** Coomassie-stained SDS-PAGE gel with standards. (**B**) Thermostability shifts of K208A in the presence of bongkrekic acid and

ADP (BKA) and carboxyatractyloside (CATR) demonstrate well folded protein. Fluorescence traces (left) and first derivatives (right). (**C**) Thermostability shift values (ΔTm) determined at 0.1-, 1- and 10-mM substrate concentrations for K208A and wild type (for comparison purposes). Circles, triangles, and diamonds represent ADP, ATP and AMP, respectively. (**D**) Capacitive currents induced by 5 mM AMP, ADP or ATP, measured by using empty proteoliposomes (no internal substrate) in which K208A was reconstituted. The curves represent one example experiment, with each current being recorded twice. (**E**) Charge movements (nC) and (**F**) normalized charge movements (using the formal charge difference between ADP and ATP), over time. Data taken from (D). (**G**) Average of normalized charge movements. The bars and error bars represent the mean and SD of two independent biological repeats. For each biological repeat, 1-3 sensor preparations were averaged, and each capacitive current was recorded twice on each sensor.
